# Biocompatibility and Antibiofilm Properties of Calcium Silicate-Based Cements: An In Vitro Evaluation and Report of Two Clinical Cases

**DOI:** 10.3390/biology10060470

**Published:** 2021-05-26

**Authors:** Maurizio Bossù, Patrizia Mancini, Erika Bruni, Daniela Uccelletti, Adele Preziosi, Marco Rulli, Michela Relucenti, Orlando Donfrancesco, Flavia Iaculli, Gianni Di Giorgio, Roberto Matassa, Alessandro Salucci, Antonella Polimeni

**Affiliations:** 1 Department of Oral and Maxillofacial Science, Sapienza University of Rome, Via Caserta 6, 00161 Rome, Italy; maurizio.bossu@uniroma1.it (M.B.); orlando.donfrancesco@uniroma1.it (O.D.); gianni.digiorgio@uniroma1.it (G.D.G.); alessandro.salucci@uniroma1.it (A.S.); antonella.polimeni@uniroma1.it (A.P.); 2 Department of Experimental Medicine, Sapienza University of Rome, Viale Regina Elena 324, 00161 Rome, Italy; patrizia.mancini@uniroma1.it (P.M.); adele.preziosi@uniroma1.it (A.P.); mr.rulli85@gmail.com (M.R.); 3 Department of Biology and Biotechnology Charles Darwin, Sapienza University of Rome, p.le A. Moro 5, 00185 Rome, Italy; erikabruni89@gmail.com (E.B.); daniela.uccelletti@uniroma1.it (D.U.); 4 Department of Anatomical, Histological, Forensic and Orthopaedic Sciences, Section of Human Anatomy, Sapienza University of Rome, Via A. Borelli 50, 00161 Rome, Italy; michela.relucenti@uniroma1.it (M.R.); roberto.matassa@uniroma1.it (R.M.); 5 Department of Neuroscience and Reproductive and Odontostomatological Sciences, University of Naples Federico II, Via Pansini 5, 80131 Naples, Italy

**Keywords:** antibiofilm properties, bioactive cements, biocompatibility, biodentine, mineral trioxide aggregate

## Abstract

**Simple Summary:**

Calcium silicate-based cements are successfully applied in the different fields of endodontics and vital pulp therapy. To better assess the properties of these bioactive materials, the present in vitro and in vivo study aimed to compare the biocompatibility and antibiofilm properties of ProRoot MTA and Biodentine. Human osteogenic sarcoma (Saos-2) cells were cultured in the presence of both materials and evaluated. Moreover, the bioactive cements were in vivo applied to perform vital pulp therapy on immature permanent teeth affected by reversible pulpitis. Saos-2 cells’ viability was slightly greater in the presence of ProRootMTA than Biodentine and cells would grow in a better way on ProRootMTA disks than on Biodentine ones. Moreover, ProRootMTA showed a powerful antibiofilm effect towards *Streptococcus mutans*. The in vitro results were clinically supported by a 100% success rate after 2 years of follow-up.

**Abstract:**

Calcium silicate-based cements have reached excellent levels of performance in endodontics, providing predictable and successful results. To better assess the properties of these bioactive materials, the present study aimed to compare the biocompatibility and antibiofilm properties of ProRoot MTA and Biodentine. Human osteogenic sarcoma (Saos-2) cells were cultured on ProRoot MTA and Biodentine samples or in the presence of both cement extracts. Cell viability assay, measurement of reactive oxygen species (ROS), immunofluorescence analysis, as well as morphological evaluations were conducted. Moreover, *Streptococcus mutans* was used to assess the biofilm forming ability on ProRoot MTA and Biodentine disks. Finally, both cements were applied in vivo to treat immature permanent teeth affected by reversible pulpitis. Results: Cell viability assay demonstrated that Saos-2 cells had a dose- and time-dependent cytotoxicity to both analyzed cements, although cells exposed to ProRoot MTA showed a better cell vitality than those exposed to Biodentine (*p* < 0.001). Both cements demonstrated ROS production while this was greater in the case of Biodentine than ProRoot MTA (*p* < 0.001). Immunofluorescence images of the cytoskeleton and focal adhesions showed no differences in Saos-2 cells grown in the presence of ProRoot MTA eluate; whereas in the Biodentine groups, cells showed a morphology and focal adhesions more similar to that of the control sample, as the eluate concentration decreased. Morphological analysis revealed that Saos-2 cells were more flattened and exhibited better spreading when attached to ProRoot MTA disks than to Biodentine ones. The antibiofilm properties showed a time-dependent powerful inhibition of *S. mutans* superficial colonization and an antibiofilm effect of both cements. Clinically, complete root formation of the treated elements was achieved using the two studied cements, showing stable results over time. ProRoot MTA and Biodentine was demonstrated to be biocompatible and to possess antibiofilm properties. Their clinical application in vital pulp therapy provided successful outcomes after 2 years of follow-up.

## 1. Introduction

Calcium silicate-based cements have achieved remarkable results in the dental field in recent years, resulting in predictable, safe, and successful therapies. Since its introduction in 1993 by Torabinejad [1], mineral trioxide aggregate (MTA) has been used in vital pulp therapy [2], in repair of root perforations or resorption, as root-end filling material in surgical endodontics [3] and, more currently, in regenerative endodontic therapy [4,5]. This hydraulic calcium silicate cement has a formulation similar to Portland cement with the addition of bismuth oxide, which is responsible for its radiopacity [6]. MTA is composed of hydrophilic particles, which, once hydrated in a wet environment, develops a hard compound consisting mainly of tricalcium silicate, tricalcium aluminate, tricalcium oxide, and silicate oxide [7]. Despite its excellent qualities, MTA has some negative aspects related to its long setting time, lower values of shear bond, difficult handling, discoloration of treated teeth in the medium/long term, and high cost [8,9]. Therefore, to overcome the aforementioned drawbacks of MTA, a new calcium silicate-based cement was introduced and applied mostly in the endodontics field, namely Biodentine. The latter is an innovative cement composed mainly of tricalcium silicate, calcium carbonate, used as a filler, and zirconium oxide, used as a radiopacifier [10]. It presents as a powder and liquid that, after mixing, hardens quickly thanks to the presence of calcium chloride in the liquid, which, on turn, reduces the setting time [11]. From a clinical point of view, Biodentine shows similar applications to MTA, such as endodontic repairs and vital pulp therapy [12]. Moreover, the same material demonstrated greater resistance to compression and flexion than other tricalcium silicate-based cements as well as sealing properties, bonding strength, and a great release of calcium ions [13,14,15]. Unlike MTA, Biodentine exhibits better handling, reduced setting time, and color stability, due to the absence of bismuth oxide, which is considered as the cause of dental discoloration [13].

On the other hand, both calcium silicate-based cements demonstrated antimicrobial activity due to their high pH and subsequent alkalinization of the surrounding environment, suggesting antibacterial potential towards several bacterial species [13,16]. In addition, they demonstrated great biocompatibility, as proven by the wide use of bioactive cements in the vital pulp therapy of deciduous teeth and permeant teeth with immature roots [6,12,17,18]. The properties of promoting mineralization as well as cell differentiation have been demonstrated to be the basis of the bioactivity of calcium silicate-based cements [6].

Therefore, the aim of the present study was to assess and compare the biocompatibility and antibiofilm activity of MTA and Biodentine. The null hypothesis was that there is no difference between the two studied cements in terms of biocompatibility and antibiofilm properties.

## 2. Materials and Methods

The present study evaluated the following materials: White ProRoot MTA (Dentsply Dental Specialties, Tulsa, OK, USA) and Biodentine (Septodont, Saint-Maur-des-Fossés, France). To better assess the materials’ features and properties, both cements were mixed according to the manufacturer’s instructions and were shaped in sterile rubber molds to obtain disks of 8 mm in diameter and 2 mm in thickness. After mixing, samples were kept in wet conditions at 37 °C for 24 h to reach complete setting. Then, the specimens were used for the morphological, cytological, and antimicrobial analyses.

### 2.1. Morphological Analysis of ProRoot MTA and Biodentine Disks

ProRoot MTA and Biodentine samples were mounted on aluminum stubs by silver glue and were observed using a variable pressure scanning electron microscope VP-SEM, Hitachi SU3500 (Hitachi, Wokingham, UK) with dual energy dispersive X-ray spectroscopy detectors (VP-SEM-dEDS), arranged in a parallel configuration (Bruker XFlash^®^ 6-60, Bruker Corporation, Billerica, MA, USA). Sample images were acquired without conductive coating at operating conditions of 5 kV and 30 Pa.

### 2.2. Cell Culture

The human osteogenic sarcoma (Saos-2) cell line, kindly provided by Prof. Riminucci, Sapienza University of Rome, was cultured in Dulbecco’s Modified Eagle’s Medium (DMEM; Euroclone, Pero, MI, Italy), supplemented with 10% fetal bovine serum (FBS; Sigma-Aldrich, MO, USA), 2 mM L-Glutamine, and 100 units/mL penicillin and 100 mg/mL streptomycin (Sigma-Aldrich, MO, USA), at 37 °C with 5% CO_2_ in a humidified atmosphere. Cells were grown to 70–80% confluency and passaged at a 1:2 ratio following trypsinization with 0.05% trypsin/0.02% EDTA (Sigma-Aldrich, St. Louis, MO, USA). Cells at passages 3–7 were used for all experiments. Cells were plated on 24-well culture plates and exposed to cement extracts, which were obtained by soaking the UV-sterilized ProRoot MTA or Biodentine disks in 2 mL of DMEM supplemented with 10% FBS kept for seven days in a humidified incubator. After this time, the cement extracts obtained were used undiluted (1:1) or diluted 1:2, 1:4, or 1:8 in DMEM and added to cells. Cells were also plated directly on the UV-sterilized disks placed on a 24-well culture plate.

### 2.3. Cell Viability Assay

Cell viability was determined by using the MTT assay as described by Ficociello et al. [19]. Briefly, 1.5 × 10^3^ Saos-2 cells were plated in 96-well plates and exposed or not to the cement extracts at 1:1, 1:2, 1:4, and 1:8 dilutions at 24, 48, and 72 h. Tetrazolium salts (MTT: 3-(4,5-dimethylthiazol-2-yl)-2,5-diphenyltetrazolium bromide (Sigma-Aldrich, St. Louis, MO, USA), 5 mg/mL suspended in PBS) were added to each well and incubated for 4 h. The formazan crystals were extracted from the cells with a solubilizing solution (DMSO). An ELISA reader (Multiskan™ FC Microplate Photometer—Thermo Fisher Scientific, Waltham, MA, USA) was used to measure the absorbance at a wavelength of 570 nm and reference length 630 nm. The results were expressed as a percentage of the viability from untreated cells. The same procedure was performed by seeding 5 × 10^4^ Saos-2 cells on Biodentine or ProRoot MTA disks and cultured for 24, 48, and 72 h, and the results are expressed as optical density values. Each experiment was performed three times in triplicate.

### 2.4. Measurement of Reactive Oxygen Species (ROS)

The intracellular reactive oxygen Species (ROS) production was analyzed as reported by Zanni et al. [20]. Briefly, Saos-2 cells, at a concentration of 3 × 10^4^, were plated on glass coverslips and exposed to ProRoot MTA or Biodentine extracts at 1:1 and 1:4 dilutions, while the negative control was obtained by seeding the same number of cells with culture medium (90% DMEM and 10% FBS) and the positive control was obtained by seeding the same number of cells with 200 µM hydrogen peroxide (H_2_O_2_). Cells were grown under these conditions for 72 h and washed twice with PBS, and subsequently incubated with fluorescent probe 2,7-dichlorodihydrofluorescein diacetate (H_2_DCFDA) (Sigma-Aldrich, St. Louis, MO, USA), 100 μM, at 37 °C for 15 min. The detection of ROS was assessed using an AxioObserver inverted microscope, equipped with the ApoTome System (Carl Zeiss Inc., Oberkochen, Germany) by evaluating the number of positive cells for ROS production as compared to the total number of counted cells.

### 2.5. Immunofluorescence Microscopy Analysis

Firstly, 3 × 10^4^ Saos-2 cells grown on glass coverslips for 72 h in the presence or not of the cement extracts at 1:1 and 1:4 dilutions were fixed with 4% paraformaldehyde for 30 min, followed by treatment with 0.1 M glycine in PBS for 30 min and with 0.1% Triton X-100 in PBS for additional 5 min, to allow permeabilization. To analyze actin cytoskeleton, Saos-2 cells were incubated with TRITC-phalloidin (Sigma-Aldrich, St. Louis, MO, USA) (1:50) for 45 min at 25 °C, as described by Vanni et al. [21]. To evaluate the adhesion capability to the cements, cells were labelled with anti-Focal Adhesion Kinase (FAK) polyclonal antibody (C-20: sc-558) (Santa Cruz Biotecnology, Dallas, TX, USA), 1:100 in PBS for 1 h, followed by goat anti-rabbit Alexa Flour 488 (Biotium, Fremont, CA, USA), 1:100 for 30 min. Nuclei were stained with 4′,6-diamidino-2-phenylindole (DAPI). Coverslips were finally mounted with Mowiol (Sigma-Aldrich, St. Louis, MO, USA) for observation.

The immunofluorescence signal of Saos-2 cells was analyzed by recording stained images using an Axio Observer Z1 inverted microscope, equipped with an ApoTome.2 System (Carl Zeiss Inc., Oberkochen, Germany). The ApoTome system provides an optical section of fluorescent samples, calculated from three images with different grid positions without a time lag. Digital images were acquired with the AxioCam MRm high-resolution digital camera (Zeiss) and processed with the AxioVision 4.8.2 software (Zeiss). ApoTome optical sectioning images of fluorescent Saos-2 cells were recorded under 40 Å~/0.75 objective (Zeiss).

Measurement of the length of focal adhesions (FAs) was determined manually by the AxioVision 4.8.2 software (Carl Zeiss Inc., Oberkochen, Germany). Further, the number of FAs per cell and the number of cells involved in FAs were analyzed. For quantitative FAs analysis, at least 25 cells on 15 pictures per replicate were analyzed.

### 2.6. VpSEM and dEDS Analysis

The disks seeded with Saos-2 cells, as previously described, were fixed with glutaraldehyde 2.5% in PBS 0.1 M pH 7.4 for at least 24 h. After washing in PBS (2 × 10 min), cells were post-fixed with osmium tetroxide 2% in distilled H2O for 1 h and 30 min [22,23]. Subsequently, samples were washed in distilled water (2 × 10 min), and then immersed for 20 min in a 1% tannic acid aqueous solution; after that, they were washed in distilled H_2_O (3 × 10 min). One drop of Uranyless (a mixture of lanthanides, Electron microscopy sciences, Hatfield, PA, USA) for 1 min was used to add contrast. Samples were dried by filter paper and one drop of an aqueous solution 10% Hilem© IL 1000 ionic liquid (Hitachi, Wokingham, UK) was added to increase the electronic conductivity. The disks were mounted on aluminum stubs by silver glue and observed under VP-SEM, Hitachi SU3500 (Hitachi, Wokingham, UK) equipped with dual energy X-ray spectroscopy (dEDS, Bruker XFlash^®^ 6-60, Bruker Corporation, Billerica, MA, USA) to perform chemical mapping [24,25,26]. After several observations, the operating conditions were set between 15 and 60 Pa and 5 kV.

### 2.7. Evaluation of Antibiofilm Capability

The Crystal Violet staining assay was used to assess the biofilm forming ability of *Streptococcus mutans* on the surface disks made of ProRoot MTA or Biodentine. *S. mutans* ATCC 25175 was grown under aerobic conditions in Breain-Heart-Infusion broth (BHI, Difco Laboratories, Detroit, MI, USA) at 37 °C under agitation. Each disk, previously sterilized through UV rays, was allocated in a single well of a 24-well microtiter plate and covered by a BHI-broth solution added with 5% sucrose and 5 × 10^6^ cells/mL of *S. mutans* overnight-grown culture. Biofilm formation was assessed following the CV method as described by Bregnocchi et al. [27]. Briefly, the samples were washed twice with sterile water and then fixed for 15 min at 65 °C. After, the staining with 0.3% CV for 15 min was performed. Several washings with sterile water were done and samples were air-dried. Finally, 96% ethanol was used to elute CV bound to teeth biofilm and the absorbance at 600 nm was then read for CV quantification. The experiment was performed in triplicates.

### 2.8. Statistical Analysis

All data are expressed as mean values with the corresponding standard deviations (SD). To determine the significant differences, ANOVA analysis, followed by Bonferroni’s test and Student’s test, was conducted by using GraphPad Prism version 5.0 (GraphPad Software, San Diego, CA, USA).

### 2.9. In Vivo Evaluation

In order to assess the clinical performances of the evaluated calcium silicate-based cements over time, 2 patients presenting deep dental decays on permanent teeth were enrolled and treated. Dental therapies were conducted at the Department of Pediatric Dentistry of Sapienza University of Rome, in accordance with the Helsinki Declaration of 1975, as revised in 2008 (59th WMA General Assembly, Seoul, Korea, October 2008). The study protocol was approved by the Ethical Committee of Sapienza University of Rome (no. 674/3714) and informed consent was obtained from all included subjects.

Two patients (1 male and 1 female, aged 6 and 7 years, respectively) reported deep carious lesions involving the mandibular first molar (3.6). Both elements presented immature root formation and reversible pulpitis (diagnosed as non-lingering pain following temperature or osmotic stimuli) and underwent vital pulp therapy to obtain apexogenesis. Pulpotomy treatments were performed by a single operator and followed the same procedure. After administration of local anesthesia (epinephrine-free mepivacaine), teeth were isolated by rubber dam and the cavities were cleaned using low-speed round bur under abundant irrigation. After caries removal and exposure of the pulp tissue, the roof of the pulp chamber was removed, and coronal pulp was amputated by high-speed diamond bur under abundant irrigation. Hemostasis was achieved using sterile cotton pellets moisturized with saline solution for a maximum of 5 min. The two included teeth were randomly treated with ProRoot MTA and Biodentine, respectively. Both cements were prepared according to the manufacturer’s instructions and were placed on radicular pulp stumps with a thickness of 2–4 mm. In the ProRoot MTA tooth, a moistened cotton pellet was placed over the material and the tooth was temporally restored by polymer-reinforced zinc oxide–eugenol cement (IRM, Dentsply DENTSPLY International Inc., Milford, DE, USA). Definitive restoration with resin composite was performed after 3 days, following removal of the cotton pellet and once the cement hardening was clinically appreciated by a dental probe. In the Biodentine tooth, after material application, a cotton pellet and a temporary polymer-reinforced zinc oxide–eugenol restoration (IRM) were placed. The tooth was permanently restored with resin composite after 3 days following the same procedure as the ProRoot MTA tooth.

Periapical radiographs were obtained immediately after pulpotomy procedures (baseline), then both elements were clinically and radiographically evaluated by a single blinded examiner after 1, 3, and 12 months, and then every year.

## 3. Results

### 3.1. VpSEM Morphological Analysis of ProRoot MTA and Biodentine Disks

**Figure 1:**
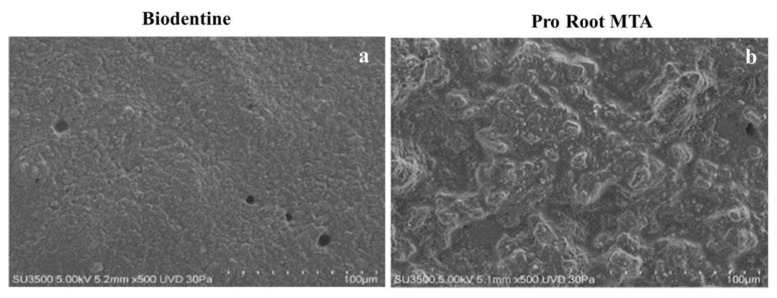
Morphological images of Biodentine and ProRoot MTA disks. (**a**) The surface of the Biodentine sample was smooth and plane, showing a fine and uniform granularity. The pores had a regular circular or elliptical shape. Magnification 500×. (**b**) In the ProRoot MTA sample, it was possible to observe a roughly irregular surface, with prominent aggregates separated by irregularly shaped depressions. The rare scattered pores had an irregular shape. Magnification 500×.

The Biodentine disk surface appeared plane and smooth, with a fine and uniform granularity. In several fields, circular- or elliptical-shaped pores could be observed (Figure 1a). On the other hand, the ProRoot MTA disk surface showed a roughly irregular appearance due to the presence of prominent aggregates, separated by irregularly shaped depressions. In few fields, rare scattered pores with an irregular shape were present (Figure 1b).

### 3.2. Effects of Cement Extracts on Cell Viability of Saos-2 Cells

**Figure 2:**
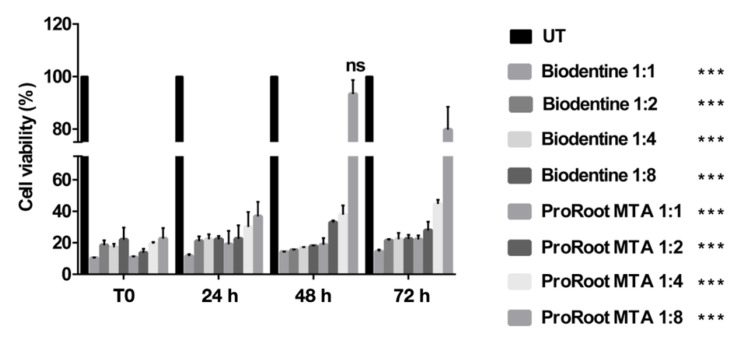
Analysis of cell viability, measured by the thiazolyl blue tetrazolium bromide (MTT) assay, of Saos-2 cells grown to serial dilution of Biodentine and Pro Root MTA extracts at 24, 48, and 72 h. Data are expressed as mean ± SD. Statistical analysis was performed by the two-way analysis of variance (ANOVA) method coupled with the Bonferroni post-test (*** *p* < 0.001 compared to the untreated sample (UT).

The MTT assay was carried out to assess the biocompatibility of Saos-2 cells exposed to serial dilutions of Biodentine and ProRoot MTA extracts. The obtained results showed that cells had a dose-dependent cytotoxicity to both analyzed cements, at any time points. In particular, cells exposed to the ProRoot MTA extract showed a better cell vitality than those grown in the presence of the Biodentine one (Figure 2). Moreover, a remarkable recovery of the vitality of the cells exposed to the lower concentration of both extracts was evident (Figure 2). Considering the MTT results, an extract dilution of 1:4 and an incubation time of 72 h were adopted for the following experiments.

### 3.3. Effects of Cement Extracts on Oxidative Stress in Saos-2 Cells

**Figure 3:**
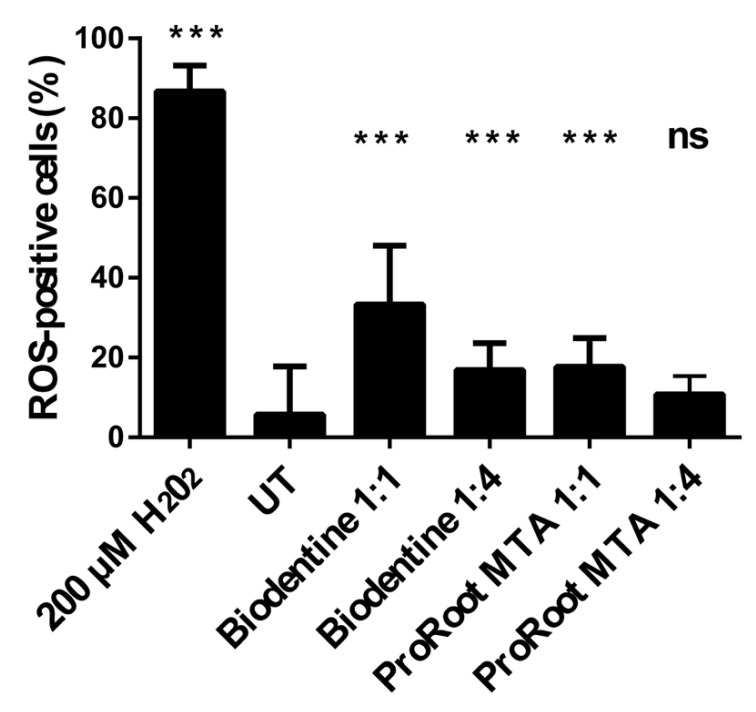
Intracellular ROS production of Saos-2 cells’ growth or not (UT) with different dilutions of extract for 72 h. Data are expressed as mean ± SD. Statistical analysis was performed by the one-way analysis of variance (ANOVA) method coupled with the Bonferroni post-test. (*** *p* < 0.001 compared to the untreated sample (UT); ns: not significant).

Oxidative stress was analyzed using the H_2_DCFDA fluorescent probe, as an indicator for ROS in the cells. The results, obtained by comparing the number of positive cells with the total number of cells, indicate that both materials were responsible for an increase of oxidative stress in Saos-2 cells in a dose-dependent manner, although this appeared higher for the case of cells exposed to Biodentine extracts with respect to those exposed to ProRoot MTA ones (Figure 3). These results suggest that the production of ROS could be the basis of different observed vitality.

### 3.4. Effects of Cement Extracts on Actin and Focal Adhesions Organization of Saos-2 Cells

**Figure 4:**
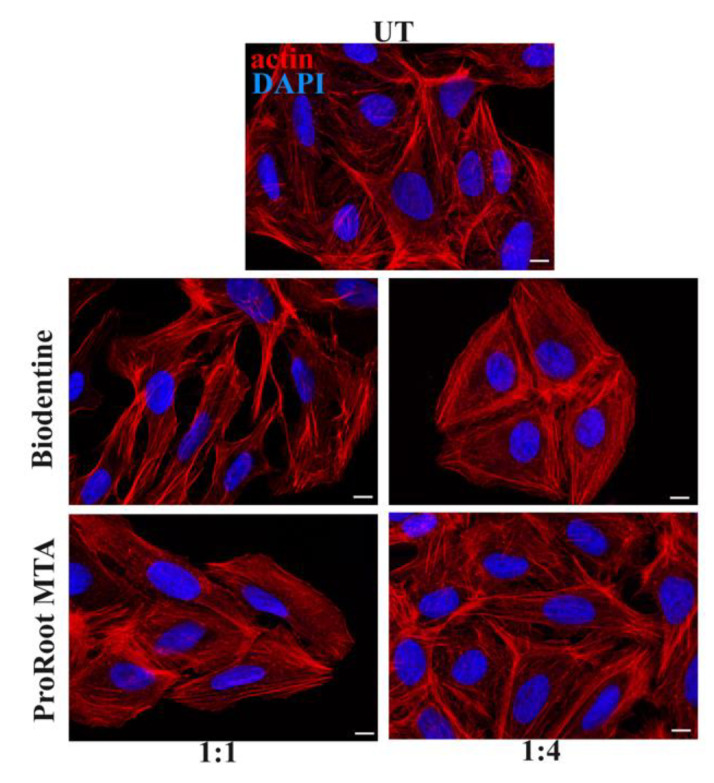
Immunofluorescence analysis of cytoskeletal actin performed on Saos-2 cells after exposure to Biodentine or ProRoot MTA extracts, respectively. UT: untreated sample. Scale bars represent 10 μm.

To evaluate the organization of the cytoskeletal actin and that of focal adhesions in Saos-2 cells, a morphological analysis was carried out by the mean of fluorescence microscopy. The analysis showed that the cells grown in the presence of the ProRoot MTA extract, at all evaluated dilutions, demonstrated their classic elongated shape, with actin mainly organized in stress fibers, and an aspect highly similar to the control cells, treated only with the culture medium (Figure 4). Whereas, in the presence of the Biodentine extract, the cells presented an altered morphology, more evident in the case of the undiluted condition (Figure 4). Moreover, in the presence of the extract at a high dilution rate, the morphology was very similar to the control sample (Figure 4).

**Figure 5:**
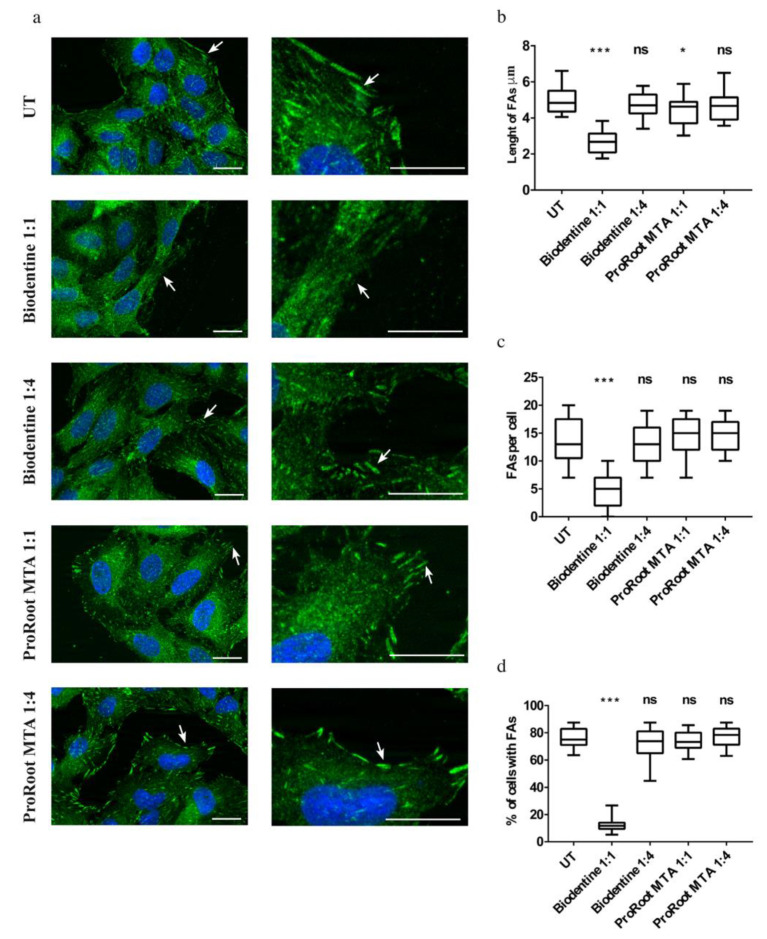
Focal adhesions (FAs) in cells. (**a**) Immunofluorescence analysis performed on Saos-2 cells labelled with anti-FAK antibodies (green) and DAPI (blue) to stain nuclei, after 72 h of treatment with Biodentine or ProRoot MTA extracts at different dilutions. Arrows indicate FAs. UT: untreated sample. Scale bars represent 10 μm. (**b**) Length of FAs, (**c**) Average number of FAs per cell and (**d**) percentage of cells with FAs. Box-and-whisker plots represent standard values (minimum, first quartile, median, third quartile, and maximum). Asterisks represent significant differences. Statistical analysis was performed by one-way analysis of variance (ANOVA) method coupled with the Bonferroni post-test (*** *p* < 0.001, * *p* <0.05) compared to the untreated sample (UT); ns: not significant).

For the adhesion capability to the two cements, morphological analysis revealed that in the presence of undiluted extract of Biodentine, the adhesion plaques were almost absent (Figure 5a). Whereas, when the eluate was less concentrated, they become more evident, although lesser in number and in size than in the presence of ProoRoot MTA. The latter group showed more evident and greater adhesion plaques than Biodentine, at all extract dilutions, providing a comparable aspect to the control sample (Figure 5a).

To confirm these morphological observations, we made a quantitative study of the FAs. The medians of the FA length in cells grown with cement extracts diluted 1:4 ranged between 4.67 μm for Biodentine and 4.7 μm for ProRoot MTA, which were similar to that of the untreated cells (4.84 μm). The median of the FA length decreased for cells grown with cement extracts from undiluted Biodentine (2.67 μm) while it increased and was similar to the control for undiluted ProRoot MTA (4.63 μm) (Figure 5b).

The lowest amount of FAs was detected on cells grown with undiluted Biodentine extract (median is 5 FAs per cell) while for Biodentine 1:4, undiluted ProRoot MTA and ProRoot MTA 1:4, medians were 13, 15, and 15 FAs per cell, respectively, similar to the control (median: 13 FAs per cell) (Figure 5c). The percentage of cells with FAs grown with cement extracts showed no particular trend, which was not significant for Biodentine 1:4, undiluted ProRoot MTA, and ProRoot MTA 1:4, while it decreased for Biodentine 1:1, as compared to the control (Figure 5d).

These results suggest a dose-dependent effect for Biodentine and a different behavior of the Saos-2 cells in terms of adhesion to the two types of evaluated cement.

### 3.5. Biocompatibility of Saos-2 Cells Cultured on ProRoot MTA and Biodentine Disks

**Figure 6:**
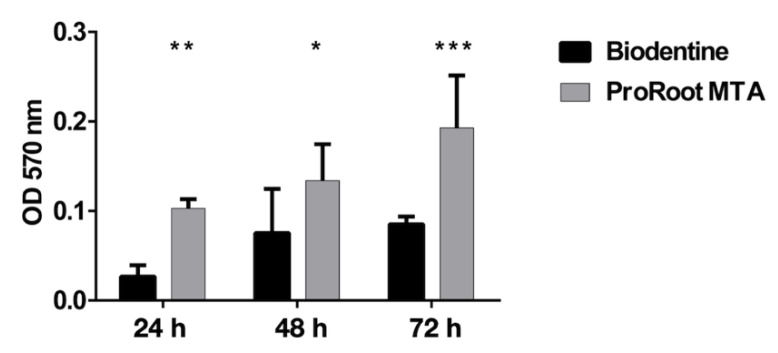
Analysis of cell viability, measured by the thiazolyl blue tetrazolium bromide (MTT) assay, of Saos-2 cells grown on Biodentine and ProRoot MTA disks for 24, 48, and 72 h. Data are expressed as mean ± SD. Statistical analysis was performed by the two-way analysis of variance (ANOVA) method coupled with the Bonferroni post-test (*** *p* < 0.001, ** *p* < 0.01, * *p* < 0.05).

To evaluate the biocompatibility of Saos-2 cells grown directly on the ProRoot MTA or Biodentine disks, an additional set of MTT assay was performed at different times. The results obtained showed that the cells were able to adhere and grow at all evaluated time points. However, cells seeded on ProRoot MTA presented a higher degree of biocompatibility compared to Biodentine (Figure 6), in agreement with the results obtained by MTT assay in the presence of both cement extracts.

### 3.6. VpSEM and dEDS Analysis of Saos-2 Cells Cultured on ProRoot MTA and Biodentine Disks

**Figure 7:**
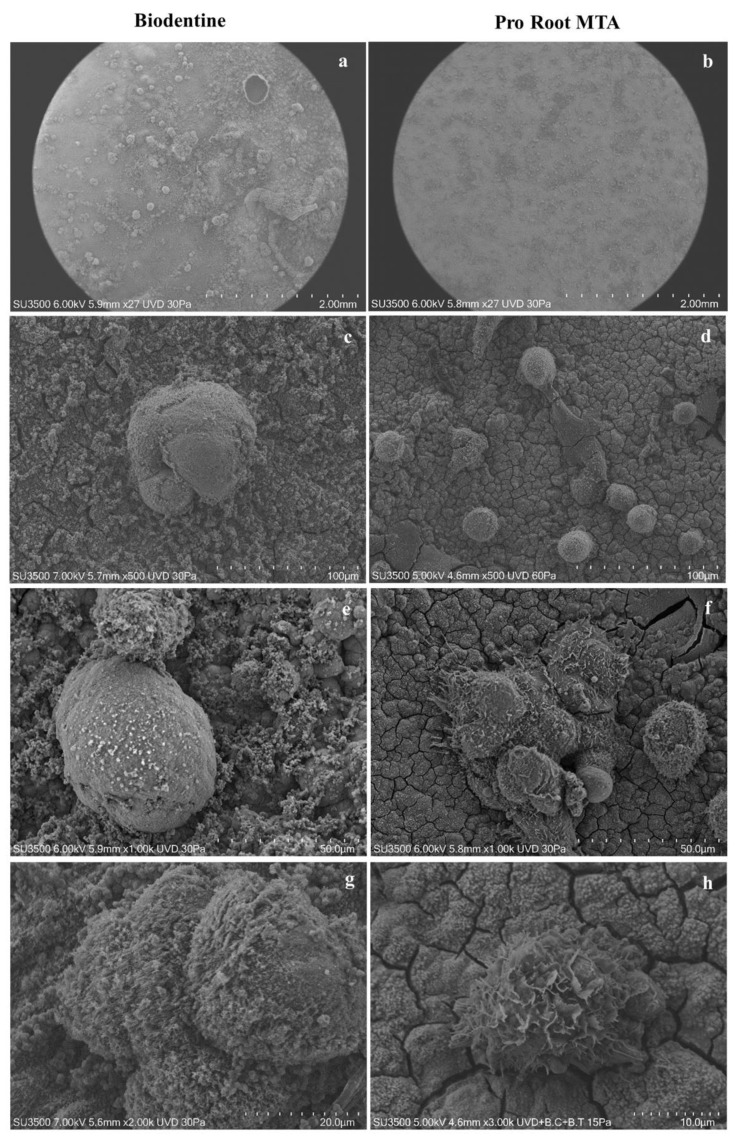
Biodentine and ProRoot and disks seeded with Saos-2 cells. (**a**) Biodentine disk, panoramic view at very low magnification. Disk surface was extremely rough and covered with spheroidal aggregates of varying sizes and uneven distribution. Magnification 27×. (**b**) ProRoot MTA disk, panoramic view at very low magnification. Disc surface appears to be dotted with a large number of evenly distributed cells. Magnification 27×. (**c**) Biodentine disk, a cluster of 4 or 5 overlapping cellular elements was visible. Cells appeared to be deformed, free of cytoplasmic projections, and partially covered with a granular coating. Magnification 500×. (**d**) ProRoot MTA disk, the individual cellular elements were visible. A round or flattened shape could be observed, with cytoplasmic projections. Magnification 500×. (**e**) Biodentine disk, an aggregate of cells with a smooth surface and remnants of microvilli could be observed. Magnification 1000×. (**f**) ProRoot MTA disk, cells were well adhered to the disk by means of filopods projecting from the basal domain. Cells presented microvilli and blebs, which could be considered as a sign of good health. Magnification 1000×. (**g**) Biodentine disk, an aggregate of cells with a smooth surface were partially covered with fine grainy material. Magnification 2000×. (**h**) ProRoot MTA disk, a single cell tightly adhered to the disc surface with well-developed lamellipods and filopods could be detected. Magnification 3000×.

At low magnification, the surface of the Biodentine disk had irregular aggregates that were considerably larger than the size of the Saos-2 cells (Figure 7a). The ProRoot MTA disk surface appeared to be dotted, in a diffuse and homogeneous manner, by a high number of Saos-2 cells (Figure 7b). By increasing the magnification, the surface of the Biodentine disks showed spheroidal aggregates formed by six to seven cellular elements (Figure 7c). Instead, on the surface of the ProRoot MTA disk, Saos-2 cells were observed as distinct, well-preserved, and spheroid-shaped entities (Figure 7d).

On the surface of the Biodentine disks, rare adherent cells could be detected, with few scattered and short microvilli on the surface of the apical domain, and numerous pluricellular aggregates. The pluricellular aggregates were formed by single cellular elements no longer attached to the material surface. These cells showed a smooth surface, without cytoplasmic extroversions; moreover, they appeared to be deformed and partially covered by a deposit of granular material. Lamellipods, pseudopods, and philopods were absent (Figure 7e–g). It could be advocated that cells seeded on the Biodentine disk’s surface were in a state of distress from moderate to severe.

On the other hand, Saos-2 cells grown on the surface of the ProRoot MTA disks expressed blebs and microvilli on the surface of the apical domain; in addition, cells appeared well attached onto the disks’ surface with their lamellipods, pseudopods, and philopods. Considering the overall morphology, it seemed that these cells were perfectly viable (Figure 7f–h).

**Figure 8:**
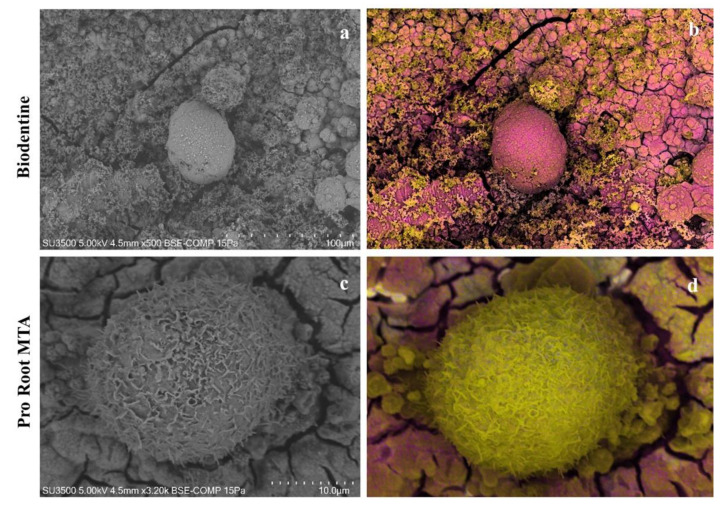
Chemical mapping of Biodentine and ProRoot MTA disks with SaoS-2 cells. (**a**) Biodentine disk, Saos-2 cell showing signs of sufferance, smooth surface, and few and short microvilli. Magnification 500×. (**b**) Biodentine disk, chemical mapping by dEDS of the same cell represented in (**a**). Cell surface is completely covered by calcium (pink). (**c**) ProRoot MTA disk, round-shaped viable Saos-2 cells showing filopods and microvilli. Magnification 3200×. (**d**) Chemical mapping by dEDS of the same cell represented in (**c**). Cell surface is rich in carbon (yellow) and calcium (pink) is only on the ground.

In order to clarify the nature of the granular material deposited on the surface of the cells observed on Biodentine disks, EDX analysis was conducted and a chemical mapping on both types of samples was performed. As shown in Figure 8, the surface of the cells seeded on the Biodentine disk was covered by a calcium deposit (pink), while the surface of the cells grown on ProRoot MTA seemed to be free of calcium deposit. On the other hand, carbon, as a constituent element of the cell membrane, was visible.

### 3.7. Antibiofilm Activity

**Figure 9:**
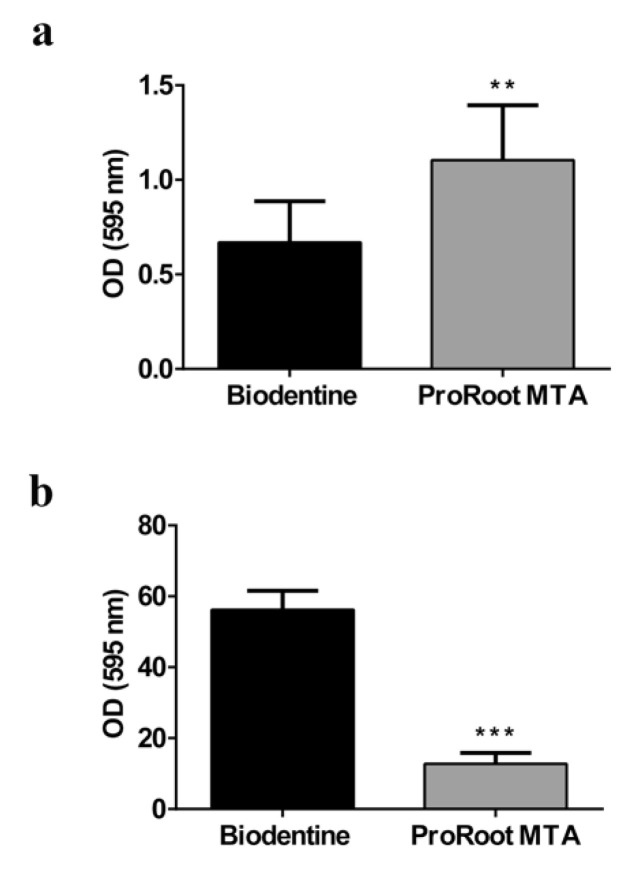
Evaluation of *S. mutans* biofilm formation on Biodentine and ProRoot MTA surface samples. Biofilm growth was evaluated by CV assay after (**a**) 4 h and (**b**) 24 h of anaerobic incubation at 37 °C. Statistical analysis was carried out using Student’s *t* test. Error bars indicate SD and asterisks indicate statistical significance (** *p* < 0.01 and *** *p* < 0.001).

Early and mature *S. mutans* biofilm formation on ProRoot MTA and Biodentine samples was evaluated. In the earliest phases, the adhesion of bacterial cells to Biodentine disks was not complete, while the surface of the ProRoot MTA samples was more covered after only 4 h of exposition (Figure 9a). After 24 h of growing, the few *S. mutans* cells adhered on Biodentine disks were able to build a mature biofilm, while on Pro-root MTA samples, their growth was inhibited, revealing a remarkable antibacterial effect with a decrease in biofilm biomass (Figure 9b). Therefore, during the initial phase, ProRoot MTA did not hinder the surface adhesion of bacterial cells, showing a great inhibition of *S. mutans*’ superficial colonization and an antibiofilm effect.

### 3.8. In Vivo Results

**Figure 10:**
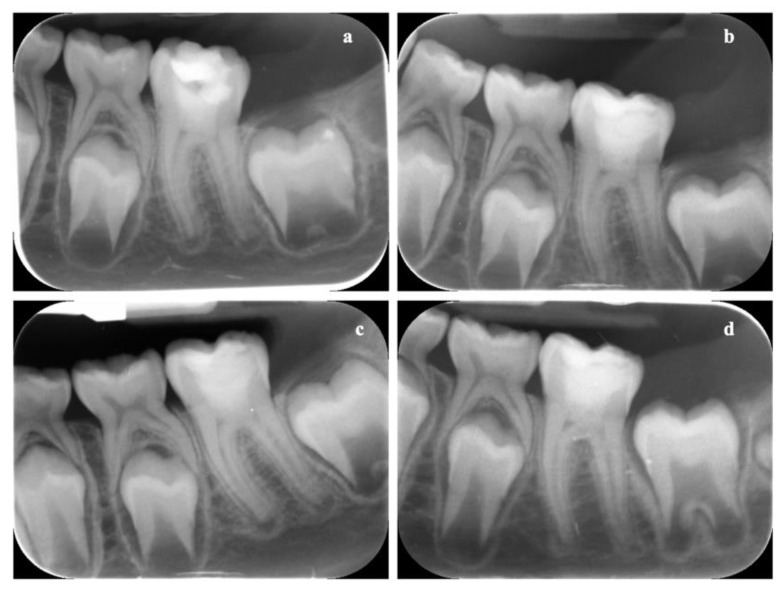
Periapical radiographs of the dental element (3.6) treated with Biodentine. The immature permanent tooth is shown immediately after vital pulp therapy (Baseline) (**a**) and after 1 month (**b**), 3 months (**c**), and 1 year of follow-up (**d**). Progressive formation of the dental roots could be observed over time with an absence of periapical reaction.

**Figure 11:**
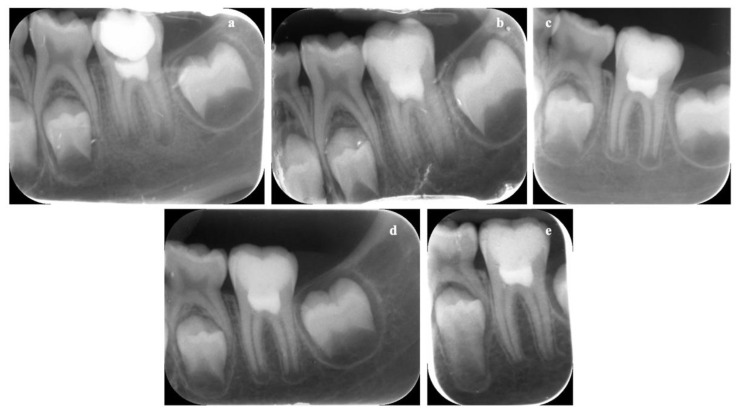
Periapical radiographs of dental element (3.6) treated with ProRoot MTA. The immature permanent tooth is shown immediately after vital pulp therapy (Baseline) (**a**) and after 1 month (**b**), 3 months (**c**), and 1 (**d**) and 2 years of follow-up (**e**). Progressive formation of the dental roots could be observed over time. Closure of apical foramina is clearly represented after 2 years of follow-up.

Clinical evaluation revealed an absence of pain, discomfort, swelling, or inflammation in both evaluated elements at every time point. The absence of pulp reaction was further confirmed by the radiographic assessment, as demonstrated by the progressive formation of the dental roots, closure of the apical foramina, as well as the increase of root wall thickness (Figure 10 and Figure 11). Stability of the clinical and radiographical situation could be observed after 1 year of follow-up in the element treated with Biodentine (Figure 10d) and after 2 years in the ProRoot MTA tooth (Figure 11e), demonstrating a success rate of 100% in both cases.

## 4. Discussion

Bioactive materials are successfully used in pulpal as well as several endodontic procedures for increasing healing outcomes [28]. These unique materials basically contain two ceramic compounds, as tricalcium silicate and dicalcium silicate, and are able to interact with water and create an alkaline pH and calcium ion release, which, in turn, induces the formation of a superficial apatite layer, demonstrating their bioactivity [28].

The sealing, antibacterial, and antifungal activities as well as the ability to either function as human tissues or encourage their regeneration [29] strengthen their promising application in medicine and dentistry [30,31]. Particularly, successful outcomes over time have been reported when bioactive calcium silicate-based cements were applied in vital pulp therapy, endodontic restoration, endodontic sealing, and regenerative endodontics [5,28,29,32]. In this respect, it is essential to confirm the materials’ biocompatibility and antibacterial properties. Therefore, the aim of this study was to assess and compare the biocompatibility and the antimicrobial properties of Biodentine and ProRoot MTA. Human osteosarcoma Saos-2 cells were used for this purpose, as they have been demonstrated to be a good model for osteoblastic function in vitro [33].

In the present study, the MTT assay was conducted to measure mitochondrial dehydrogenase activity in living, metabolically active cells in order to evaluate the biocompatibility of Biodentine and ProRoot MTA in Saos-2 cells. This assay was also performed because both materials are hydrophilic and likely to release ionic components that would be more apt to interfere with intracellular enzyme activity [34]. Furthermore, a series of extracts of different material concentrations were made to observe a possible dose–response relationship in cells and to simulate an immediate post-surgical condition [35]; interestingly, Saos-2 cells exposed to the extract of Biodentine and ProRoot MTA revealed a dose-dependent cytotoxicity to both cements used, at any time analyzed. In particular, cells exposed to ProRoot MTA extract showed a similar cell vitality to the control group at the 1:4 and 1:8 dilutions, and a better vitality than those grown in the presence of the Biodentine extract. Zanini et al. [36] noticed similar results with a significant decrease in murine pulp cells proliferation, 2 days after stimulation with Biodentine; the authors speculated that it could be caused by the release of calcium hydroxide from the material and by pH increase. A similar result was obtained with human dental pulp stem cells (hDPCs) exposed to Biodentine by Luo et al. [37]. On the other hand, Pelliccioni et al. [38] reported how Saos-2 cells, challenged with ProRoot MTA for 24 and 72 h, showed a better behavior than cells exposed to other compounds under assay. According to MTT assay, the dilution of 1:1 and 1:4 was selected to investigate if the different biocompatibility could be attributed to ROS accumulation caused by exposure to the two materials. In fact, the evaluation of intracellular ROS formation is important to understand cellular responses to the test materials. ROS are a natural product of normal oxygen metabolism and have been found to play important roles in cell signaling, proliferation, and survival [39]. Experimental evidence on various materials suggests that the cell toxicity mechanism is often attributed to the ability of these materials to induce oxidative stress with ROS accumulation [40]. To explain the different biocompatibility obtained using extracts from Biodentine and ProRoot MTA, we wondered if in our experimental system the change of vitality could also be attributed to the induced oxidative stress caused by exposure to the two materials. Our results showed a higher ROS production in samples exposed to the Biodentine eluate than the ProRoot MTA one, probably because it might influence the cell vitality. This result was in agreement with the MMT assay and was confirmed by the morphological analysis of Saos-2 cells. Indeed, in the presence of undiluted elute of Biodentine, the cells exhibited altered morphology in actin stress fibers, which became more similar to those grown in the normal condition, where the eluate was more diluted.

According to the scientific literature [41], in the present study, it was demonstrated that Saos-2 cells grew in a better way on ProRoot MTA disks than on Biodentine ones; this was due to the different surfaces’ morphology, since ProRoot MTA showed the roughest surface and a rounded granule micromorphology. In addition, the MTT assay results indicated that the viability of cells grown on ProRoot MTA disks was higher than those grown on Bioedentine, while ROS amounts were higher in Saos-2 cells on Biodentine disks than on ProRoot MTA ones. The morphology of Saos-2 cells perfectly reflected these biochemical findings; indeed, Saos-2 cells grown on ProRoot MTA disks appeared as spheroid-shaped entities with a well-preserved surface, blebs, and microvilli on the surface of the apical domain. Cells adhered to each other and to the substrate due to integrins and the normally functional cytoskeleton, whose integrity was also confirmed by the presence of lamellipods, indicative of cell spread. This morphology was the same as the cells cultured only in DMEM medium used as a control in the study conducted by Ayobian-Macarazi et al. [42]. Moreover, Saos-2 cells grown on Biodentine disks were covered by calcium, as demonstrated by dEDS analysis. Calcium ions are fundamental second messengers, easing cells’ attachment to substrates and cell motility, as well as modulating the function of cadherins, selectins, and integrins [43,44] and cell–cell interactions. Calcium ions are also involved in metabolic processes [45,46] and signal transduction [47,48]. When the extracellular concentration of calcium ions is too high, mitochondria cannot maintain the normal intracellular concentration of calcium ions. This may cause some consequences, such as interruption of ATP synthesis, depolarization of the inner-mitochondrial membrane, increase of mitochondrial matrix pH, and complete and irreversible opening of the mitochondrial permeability transition pore, which induces a cascade of events that lead to apoptosis [49]. Cells grown on Biodentine disks showed a typical morphology of apoptotic or severely distressed cells. Probably, the calcium ion concentration allowed cells to recover their ability and induced cytoskeleton rearrangement, resulting in cell detachment, and destruction of cellular lamellipods and microvilli, in a sequence of a signaling cascade that might end with apoptosis.

Overall, these results led us to hypothesize that the difference in biocompatibility and morphology could be due to a different kind of adhesion, which depends on the substrate on which cells grow, according to the components of the substrate and also to the cell type. The composition of the cellular matrix on which cells grow in vivo determines the cell–substrate and cell–cell adhesion characteristics. These can be recreated in vitro through the use of particular matrices, and nanomaterials can also regulate these interactions. In this report, we evaluated the response of the Saos-2 cells in terms of adhesion towards the two materials used and analyzed the adhesion protein FAK both from a morphological and quantitative point of view.

As expected, at the time of our observations (72 h), cells appeared well adhered to the substrate, with large adhesion plaques localized especially along the periphery of the cells; however, those grown in the presence of undiluted Biodentine showed very few small plaques. This could be explained by the fact that Biodentine could release calcium ions, which interfere with the chemical bonds responsible for correct adhesion. In fact, calcium plays a very important role; for example, in the case of cadherins, molecules are responsible for cell–cell interaction [50], or integrins, which regulate cell–substrate interaction [51]. These morphological data were confirmed by the quantitative analysis, carried out as reported by Peterková et al. [52,53], indicating a dose-dependent effect for Biodentine, and a different behavior of the Saos-2 cells in terms of adhesion to the two types of evaluated cements.

The antibiofilm and antibacterial activity of both materials were assessed using *S. mutans*, as it has largely been demonstrated to be strongly related to caries incidents due to its pathogenesis [54] and its capability to form robust biofilms on human teeth [55]. The outcomes obtained in the present study demonstrated an initial surface adhesion of bacterial cells on ProRoot MTA, whereas after 24 h, the same material showed a powerful inhibition of *S. mutans* superficial colonization and an antibiofilm effect. On the contrary, at the same time point, adherent *S. mutans* cells able to build a mature biofilm were detected on Biodentine disks, although the bacterial count was limited. These results are in agreement with previous studies evaluating the antibacterial effects of both cements toward *S. mutans* [56,57], even though Poggio et al. [56] reported any bacterial growth inhibition of Biodentine.

Finally, the report of the two clinical cases showed that both cements yielded successful clinical results when applied in vital pulp therapy of immature permanent teeth. After 1 and 2 years of follow-up, respectively, it might be speculated that apexogenesis, as the continued physiologic development and formation of the root’s apex [58], was obtained.

As reported by Sequeira et al. [59], MTA and Biodentine demonstrated good biocompatibility, promoting mineralized dentinal tissue deposition when applied in vivo. The authors showed a greater amount in the presence of Biodentine and underlined the induction of new hard tissue formation when bioactive materials were applied.

Although the present study had a limited sample size, the clinical application of the evaluated materials was reported to partially overcome the discrepancies between the in vitro and in vivo results, considering that the clinical oral environment might influence the performances of both cements. However, further studies should be conducted to support these outcomes with a larger sample size and longer follow-up period.

## 5. Conclusions

Within the limitation of the present study, it can be concluded that both cements are biocompatible, although ProRoot MTA provided a cellular viability greater than Biodentine, and that Saos-2 cells have a different behavior in terms of adhesion towards the two types of evaluated cements, suggesting a dose-dependent effect for Biodentine. Moreover, the clinical application of both cements in vital pulp therapy supported the in vitro results, demonstrating a 100% success rate after 2 years of follow-up.

## Data Availability

The data presented in this study are available on request from the corresponding author.
